# Dissecting Oct4 enhancer function in pluripotent stem cells and mouse embryogenesis

**DOI:** 10.1016/j.stemcr.2025.102705

**Published:** 2025-11-06

**Authors:** Lawrence E. Bates, Jennifer Nichols

**Affiliations:** 1 MRC Human Genetics Unit, Institute of Genetics and Cancer, University of Edinburgh, Western General Hospital, Crewe Road South, Edinburgh EH4 2XU, UK

## Main text

In this issue of *Stem Cell Reports*, Schmitz, Okamura et al. manipulate enhancer elements of the endogenous Oct4 locus to investigate consequences on Oct4 expression and cell fate. While their findings confirm some previous results, they also identify interesting discrepancies from earlier studies using conventional reporters or total Oct4 knockouts.

Oct4 is a critical transcription factor in vertebrate development. It was identified as an expressed factor in pluripotent cell populations in the mouse preimplantation, early post-implantation embryo, and primordial germ cells (Scholer et al., 1990). Given the facility with which germ cells could be converted to pluripotent embryonic germ cells in culture (Resnick et al., 1992) and generate teratocarcinomas *in vivo*, it was speculated that Oct4 might be a master regulator of the capacity for multilineage differentiation. Subsequently, Oct4 was found to be both necessary and refractory for the establishment and maintenance of naive (preimplantation-like) pluripotency in mouse (Niwa et al. 2000; Radzisheuskaya et al., 2013; Karwacki-Neisius et al., 2013). Conditional knockout of Oct4 in the mouse post-implantation epiblast results in disorganized morphology, with axis disruption, a failure to progress beyond the onset of gastrulation with complete failure to develop anatomical features by embryonic day 8.5 (Mulas et al., 2018).

To investigate the regulation of Oct4, transgene reporters were developed using regions upstream of the Oct4 transcriptional start site to drive the expression of LacZ or GFP, along with a series of internal deletions (Yeom et al., 1996; Yoshimizu et al., 1999). An 18 kb fragment (GOF-18) appeared to reproduce the pattern of Oct4 expression observed during pluripotency and germ cell development. A small internal deletion overlapping the promoter region resulted in total loss of expression, whereas removal of a neighboring proximal enhancer element had little impact on reporter activity in embryonic stem cells (ESCs), preimplantation embryos, or primordial germ cells (Yoshimizu et al., 1999; Yeom et al., 1996), but reporter activity was downregulated and lost prematurely in the post-implantation epiblast and post-implantation-like human ESCs and mouse epiblast stem cells (EpiSCs) (Yoshimizu et al., 1999; Theunissen et al., 2014; Tesar et al., 2007). Meanwhile, deletion of a more distant region, a distal enhancer (DE) element, had minimal impact on reporter expression in the post-implantation epiblast or EpiSCs but prevented expression in preimplantation embryos and mouse ESCs (Tesar et al., 2007; Yoshimizu et al., 1999).

However, there are drawbacks to this reporter approach. The genomic location of integration may have an impact on expression and regulation. More distant regulatory elements may be missing from these reporters. Such reporters must develop their epigenetic state *de novo* and consequently may establish a distinct profile from the endogenous locus. As expected, disruption of the proximal enhancer element, critical for post-implantation Oct4 expression, was found to have minimal impact on mouse blastocysts. Mouse ESCs with this endogenous ΔPE modification were competent for multilineage differentiation but experience a high rate of apoptosis, indicating a critical role for Oct4 in the survival of cells exiting pluripotency. Accordingly, the authors found that ΔPE ESCs injected into wild-type blastocysts were only minimally capable of contributing to chimeras by later stages of gastrulation, with very limited contribution to live-born pups. Interestingly, the authors found that ΔPE cells continued to express Oct4 for the first few days of *in vitro* differentiation, during a period of transition through a transient formative state, prior to loss of Oct4 and inability to maintain pluripotency as they advanced to a primed identity.

The *in vivo* requirement for the DE, known to be essential for preimplantation expression of Oct4, was investigated using ΔDE embryos generated by inter-crossing heterozygous mice. Despite the enhancer deletion, they observed the expression of Oct4 in the early blastocyst at E3.5, contrary to previous reports using a ΔDE reporter construct in which reporter activity was not observed until after implantation (Yoshimizu et al., 1999). This suggests that more distant regulatory regions, or complex epigenetic regulation of the endogenous Oct4 locus not captured by exogenous reporter assays, may be critical for Oct4 regulation prior to segregation of the inner cell mass into epiblast and extraembryonic endoderm and establishment of the naive pluripotent compartment.

These findings highlight the importance of manipulating endogenous genomic elements within their natural context for full understanding of their function. The approach the team took in this work will surely be extended to implementation of conditional knockouts in the future in order to study the function of different enhancer elements at later developmental stages, for example, the role of the endogenous distal enhancer of Oct4 in primordial germ cell development. Such advances would also facilitate the study of Oct4 enhancer elements in human naive pluripotent stem cells and in models of human development such as gastruloids. Moreover, these same techniques will be directly applicable to studies of the regulation of additional genes, including other development-associated factors and those associated with enhanceropathies, which have an increasingly recognized role in disease.

## Declaration of interests

The authors declare no competing interests.
